# CAP2 in cardiac conduction, sudden cardiac death and eye development

**DOI:** 10.1038/srep17256

**Published:** 2015-11-30

**Authors:** Jeffrey Field, Diana Z. Ye, Manasi Shinde, Fang Liu, Kurt J. Schillinger, MinMin Lu, Tao Wang, Michelle Skettini, Yao Xiong, Angela K. Brice, Daniel C. Chung, Vickas V. Patel

**Affiliations:** 1 Department of Systems Pharmacology and Translational Therapeutics, University of Pennsylvania Perelman School of Medicine Philadelphia, Pennsylvania 19041 USA; 2 Cardiovascular Institute, University of Pennsylvania Perelman School of Medicine Philadelphia, Pennsylvania 19041 USA; 3 Section of Cardiac Electrophysiology, University of Pennsylvania Perelman School of Medicine Philadelphia, Pennsylvania 19041 USA; 4 University Laboratory Animal Resources and School of Veterinary Medicine, Department of Pathobiology, University of Pennsylvania, Philadelphia, Pennsylvania; 5 Scheie Eye Institute, University of Pennsylvania Perelman School of Medicine, Pennsylvania 19041 USA.

## Abstract

Sudden cardiac death kills 180,000 to 450,000 Americans annually, predominantly males. A locus that confers a risk for sudden cardiac death, cardiac conduction disease, and a newly described developmental disorder (6p22 syndrome) is located at 6p22. One gene at 6p22 is CAP2, which encodes a cytoskeletal protein that regulates actin dynamics. To determine the role of CAP2 *in vivo*, we generated knockout (KO) mice. *cap2*^−^/*cap2*^−^ males were underrepresented at weaning and ~70% died by 12 weeks of age, but *cap2*^−^/*cap2*^−^ females survived at close to the expected levels and lived normal life spans. CAP2 knockouts resembled patients with 6p22 syndrome in that mice were smaller and they developed microphthalmia and cardiac disease. The cardiac disease included cardiac conduction disease (CCD) and, after six months of age, dilated cardiomyopathy (DCM), most noticeably in the males. To address the mechanisms underlying these phenotypes, we used Cre-mediated recombination to knock out CAP2 in cardiomyocytes. We found that the mice developed CCD, leading to sudden cardiac death from complete heart block, but no longer developed DCM or the other phenotypes, including sex bias. These studies establish a direct role for CAP2 and actin dynamics in sudden cardiac death and cardiac conduction disease.

Sudden cardiac death is a leading cause of mortality and about 20% of those affected have an underlying cardiomyopathy^1^. In patients without coronary artery disease, the highest risk of sudden death occurs among males with cardiomyopathy and intraventricular conduction delay. Dilated cardiomyopathy (DCM) is the most common form of non-ischemic cardiomyopathy. It occurs more frequently in men than in women, but the sex bias towards males is poorly understood and few animal models with DCM show a sex bias^2^,^3^,^4^. DCM can be preceded by conduction disease, including heart block^5^,^6^. The region surrounding 6p22 contains a locus involved in cardiac and developmental disorders. Genome wide association studies (GWAS) mapped loci between 6p21 and 6p22.3 associated with sudden death, hypertension, cardiac conduction, left ventricular hypertrophy, coronary heart disease and height^7^,^8^,^9^,^10^,^11^. Additionally, a rare developmental disorder defined by deletions in the distal part of the short arm of chromosome 6 was recently identified and named 6p22 syndrome. 6p22 patients are characterized by developmental delays, autism spectrum disorders and various degrees of penetrance of the following: anterior eye chamber abnormalities, hearing loss, ear abnormalities, micrognathia, hypotonia and heart defects^12^,^13^,^14^,^15^. However, little is known about most of the genes in this region. CAP2 is deleted in almost all of the 6p22 patients^16^.

CAP2 is part of the actin cytoskeleton, which regulates cell shape, cell motility and muscle contraction. The cytoskeleton is assembled by polymerization of globular actin (G-actin) monomers into filamentous actin (F-actin). The balance of F-actin and G-actin is coordinated by actin binding proteins. Mutations in cardiac cytoskeletal proteins typically cause congenital cardiac problems, most commonly dilated cardiomyopathy (DCM)^3^,^17^.

CAP was first found in yeast as a subunit of adenylyl cyclase^18^ and, independently, in yeast genetic screens for components of Ras/cAMP signaling^19^,^20^. However, cyclase binding is only found in yeast so it will not be discussed further^21^. Notably, all CAP homologs regulate the cytoskeleton by binding G-actin and cofilin^22^,^23^. Microinjection of antibodies suggested a role for CAP1 in regulating actin filaments^24^ and knocking down CAP1 causes extensive actin filaments that turn over slowly^25^. The effects of CAP on filament turnover require cofilin, which directly accelerates actin filament turnover. Although CAP has little effect on filament turnover *in vitro*, it accelerates cofilin-promoted turnover and filament severing^26^,^27^,^28^,^29^.

CAPs are conserved in all eukaryotes. In *Drosophila*, CAP mutations cause eye problems by affecting the formation of the morphogenetic furrow in the eye imaginal disc. Similar problems are seen in other cytoskeletal mutants and are a consequence of F-actin accumulation causing premature hedgehog and hippo-induced photoreceptor differentiation^30^,^31^. In *C. elegans*, CAP mutations cause defects in sarcomeric actin organization^32^ and knockdown of CAP2 in zebrafish causes a short-body phenotype and pericardial edema^33^.

There are two CAP homologs in mammals, CAP1 and CAP2. CAP1 is widely expressed in most cells and tissues^25^, while CAP2 expression is restricted to the brain, skin, skeletal muscle, cardiac muscle and testis^25^,^34^. At the subcellular level, CAP2 is found in the cytoplasm although, unlike other isoforms, some CAP2 exists in the nucleus. In adult muscle, CAP2 localizes to the M-line of sarcomeres with myomesin, which attaches myosin thick filaments to the M line^34^. In skin, CAP2 is present at the nuclear membrane and also at cell-cell junctions^34^. Since at least one of the two CAP isoforms is expressed in nearly all cells, it is likely that CAP1 and CAP2 complement each other in some cellular functions, but CAP2 may have unique roles, especially in skeletal and cardiac muscles^25^,^34^.

To determine the function of CAP2 *in vivo*, we created whole body and conditional knockout mice. Whole body deletion of CAP2 reduced survival in males, with few surviving to weaning and about 70% of the survivors dying suddenly between 5 and 10 weeks of age. Surviving males developed dilated cardiomyopathy by 12 months of age. Mice with cardiomyocyte-specific deletion of CAP2 also died suddenly due to complete heart block. CAP2 knockout mice are also prone to eye infections, the underlying cause of which appeared to be microphthalmia. These studies show that CAP2 knockout mice may be useful for understanding the mechanisms of sudden cardiac death in some cardiomyopathies, studying the role of the actin cytoskeleton in the cardiac conduction system and modeling some of the phenotypes in 6p22 patients.

## Results

### Generation CAP2 knockout and conditional knockout mice

**Figure 1:**
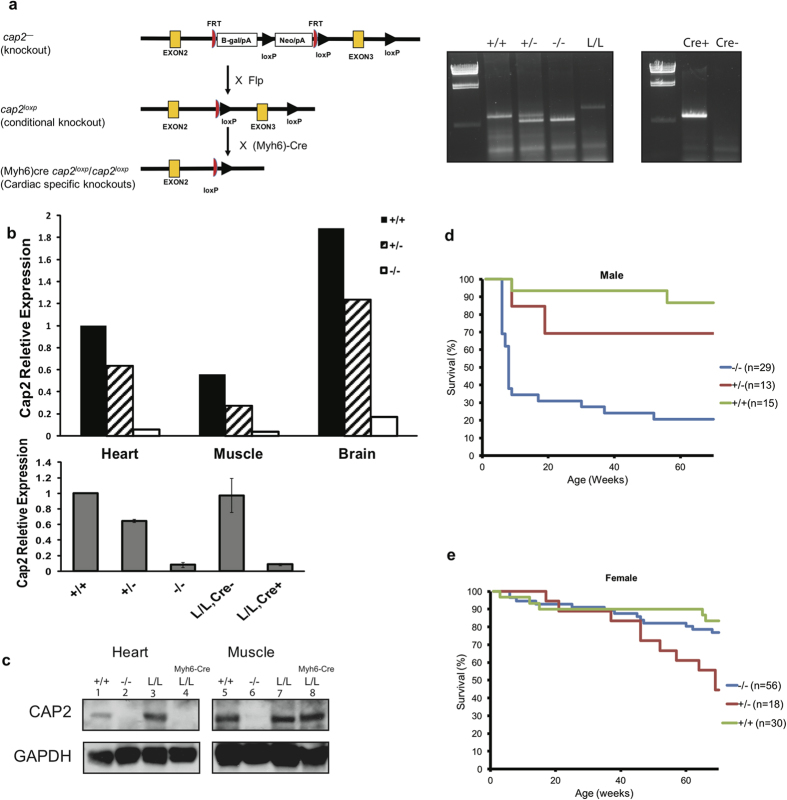
CAP2 knockout strains and survival curves. (**a**) the strategy for creating the CAP2 knockout mice used in this study and an example of a genotyping gel, (**b**) upper panel, expression of CAP2 in heart, muscle and in brain analyzed by qPCR; lower panel, expression in hearts analyzed by qPCR. The ages of mice in the upper panel are 3 wks. Three mice of varying ages were used for each genotype in the lower panel. Although the ages ranged from 3 weeks to 69 weeks, expression levels did not change significantly between mice of the different ages (**c**) Western blots of CAP2 in heart and muscle. (**d**) Kaplan-Meier survival curve for males (**e**) Kaplan-Meier survival curve for females. The genotypes are: wild type (+/+), whole body knockout (cap*2*^−^/cap*2*^−^ or −/−), rescued mice (cap2^loxp^/cap2^loxp^ or L/L) and cardiac specific knockouts (Myh6Cre-cap2^loxp^/cap2^loxp^ or L/L, Cre+). Error bars ± S.E.M.

To determine the role of CAP2 in mice, we knocked out CAP2 in C57/B6 mice by insertional mutagenesis. The European Conditional Mouse Consortium (EUCOMM) generated the targeting construct and ES clones. Figure 1a shows the strategy for creating three transgenic genotypes, *cap2*^−^, *cap2*^*loxp*^ and *Myh6Cre*-*cap2*^*loxp*^/*cap2*^*loxp*^ as well as an example of a genotyping experiment. The *cap2*^−^ construct introduced an insert within intron 2 that results in a truncated fusion protein containing 40 amino acids of CAP2. The construct also introduced FRT sites flanking the insert and loxP sites flanking exon 3. Crossing *cap2*^−^ mice to actin-FLP mice to create *cap2*^*loxp*^ deleted most of the insert and restored CAP2 expression, but left the two loxP sites flanking exon 3. Crossing the *cap2*^*loxp*^ mice to appropriate Cre-recombinase expressing mice creates tissue-specific deletions of CAP2. Deletion of the *cap2* gene was tested by extensive PCR mapping of the ES cell lines. To analyze expression, we extracted RNA from heart, muscle and brain of wild-type controls (+/+), heterozygotes (+/−) and knockout mice (−/−), and performed q-PCR reactions (Fig. 1b upper panel). *cap2* expression was significantly reduced in all three tissues in the −/− mice, while there was a partial loss of expression in heterozygotes. Additionally, crossing *cap2*^*loxp*^/*cap2*^*loxp*^ with the Myh6Cre reduced the message in the heart to knockout levels (Fig. 1b, lower panel). We also tested the expression of CAP2 in heart, muscle and brain on western blots probed with a polyclonal antibody (Fig. 1c; data not shown for brain). CAP2 protein levels were diminished in *cap2*^−^/*cap2*^−^ mice, restored in *cap2*^*loxp*^/*cap2*^*loxp*^ mice and not detected in the hearts *Myh6Cre*-*cap2*^*loxp*^/*cap2*^*loxp*^ mice (Fig. 1c).

### CAP2 KO mice are smaller and males have lower survival rates.

We first assessed the growth and survival of CAP2 knockout mice. In crosses of heterozygous males with heterozygous females, the *cap2*^−^/*cap2*^−^ mice were born viable, but with fewer male mutants surviving to genotyping (~3 weeks) than females. Of 115 pups born from *cap2*^−^/*cap2*^*+*^ × *cap2*^−^/*cap2*^*+*^ crosses, only 5.3% of male *cap2*^−^/*cap2*^−^ mice survived compared to 16.9% of the female *cap2*^−^/*cap2*^−^ mice (Table 1). The numbers of heterozygote and wild-type mice born were close to the expected Mendelian ratios. In addition, the male mutants had lower survival rates compared to wild-type mice. About 70% of *cap2*^−^/*cap2*^−^ males died within 12 weeks of birth, while 5.4% of *cap2*^−^/*cap2*^−^ mutant females died by the same age (Fig. 1d,e). Heterozygote males also showed reduced survival, but started dying at about 10 weeks of age (Fig. 1d). Thus, at young ages, there was a marked sex bias in survival, with females surviving at about wild-type levels, while only about 30% of males survive to adulthood. We found that *cap2*^−^/*cap2*^−^ females were fertile when crossed to *cap2*^−^/*cap2*^−^ males or to *cap2*^−^/*cap2*^*+*^ males, but few pups survived and we were unable to maintain a colony of *cap2*^−^/*cap2*^−^ mice.

**Figure 2:**
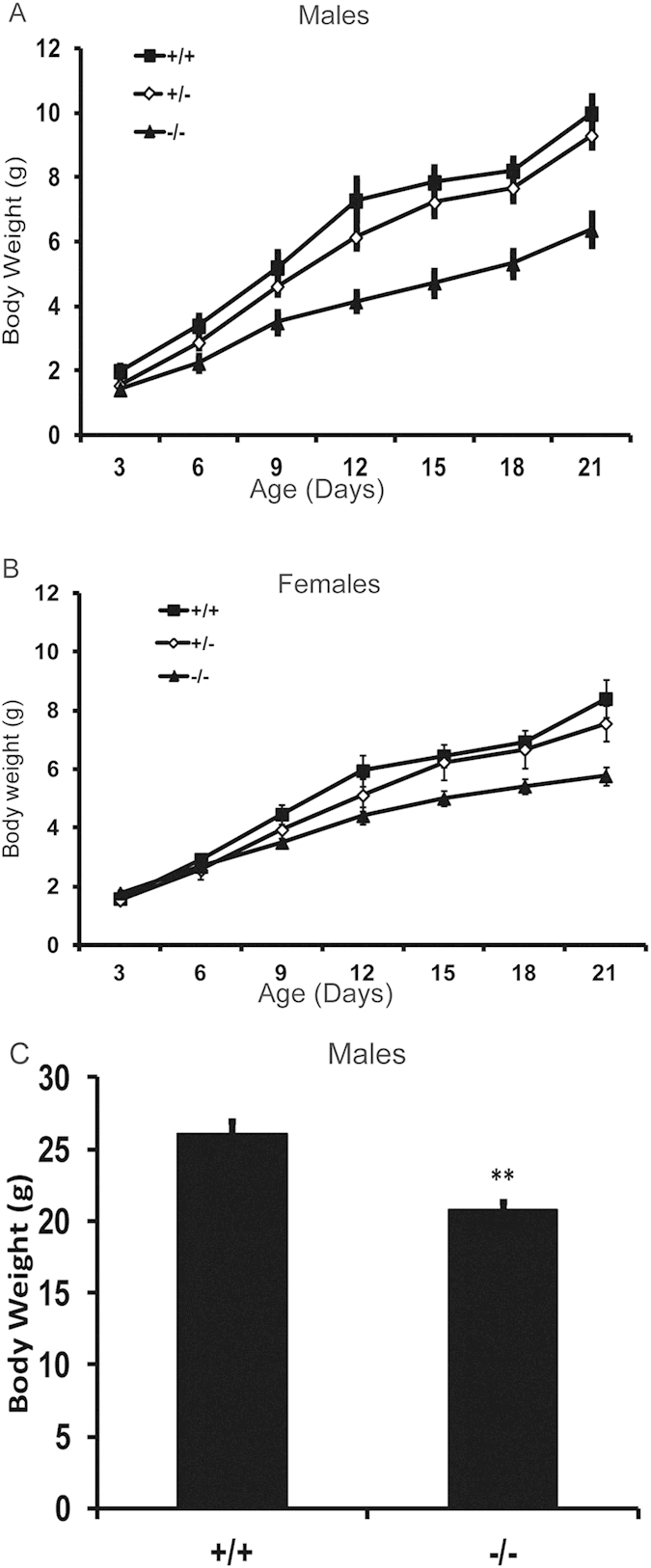
Effects of CAP2 knockout on animal growth. (**a**) Growth of male pups; (**b**) Growth of female pups; (**c**) Adult male body weight. Error bars ± S.E.M.

We also monitored the weight of male and female *cap2*^−^/*cap2*^−^ mutants, heterozygotes and littermate controls for three weeks (Fig. 2). Both male and female *cap2*^−^/*cap2*^−^ had similar weights as other groups at birth, but they grew at significantly slower rates than heterozygotes and wild-type controls. For male mutants, this trend was even more pronounced and their weight was significantly lower as early as postnatal day 3. By postnatal day 21, both male and female mutants were still smaller than age-matched heterozygotes and wild-type controls. To address if the stunted growth had a metabolic cause we performed Whole Body Dual X-Ray Absorptiometry (DEXA), but did not observe differences (Supplementary Figures S1a and S1c). Mutants were weaker in grip strength assays (Supplementary Figure S1b), but the differences were probably a reflection of their smaller size. We did not observe any abnormalities in isolated EDL muscles when analyzed by electron microscopy (data not shown). The mutants were normal for glucose (Supplementary Figure S1d) and the utilization of fuels by the respiration exchange ratio (RER) (Supplementary Figure S2).

### CAP2 deletion causes eye developmental defects.

**Figure 3:**
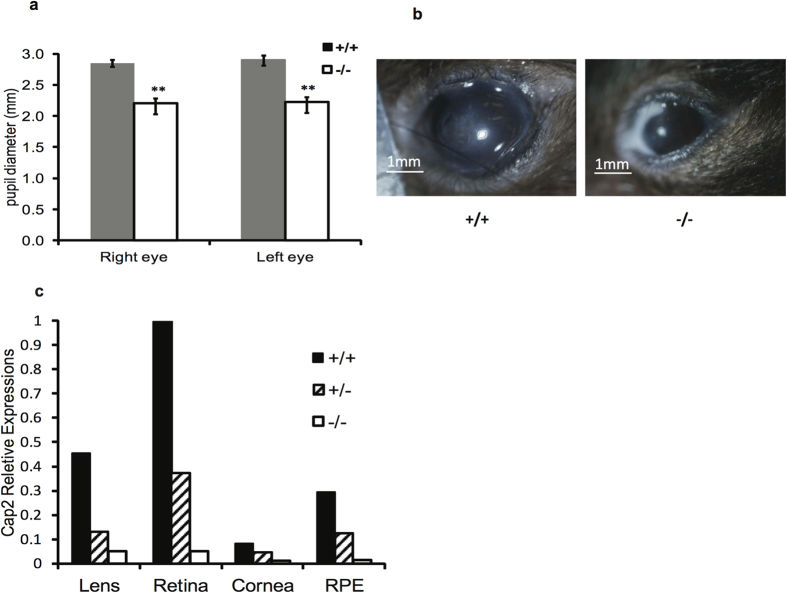
Eye infections and microphthalmia in knockout mice. (**a**) Pupil diameter in *cap2*^−^/*cap2*^−^ and *cap2*^*+*^/*cap2*^*+*^ mice. Error bars ± S.D. (**b**) Examples of eye infections seen in *cap2*^−^/*cap2*^−^ mice. Photos by DCC. (**c**) CAP2 expression in lens, retina, cornea and retinal pigment epithelium (RPE) was determined by qPCR.

We found that knockouts were prone to eye inflammation and infections, which were observed in almost all males and about half of the females. Infections typically appeared at about 6 weeks of age and were more common in the right eye than the left eye. To address if infections were associated with vision problems, we performed electroretinography and pupillometry but did not find any significant differences from normal mice (data not shown). However, the diameters of pupils in male mutants were significantly decreased, suggesting an underlying eye development problem such as microphthalmia (Fig. 3a,b). Expression was detected in several regions of the eye and was highest in the retina and lens (Fig. 3c)^35^.

### Cardiomyopathy in CAP2 mutants

**Figure 4:**
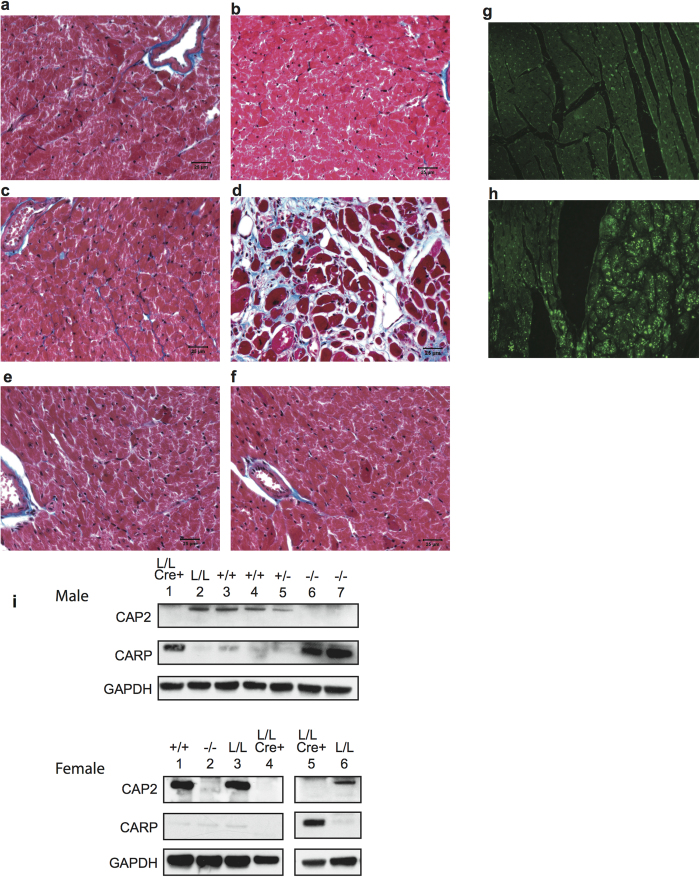
DCM in older male mice. (**a**–**f**) Trichrome staining of adult hearts (**a**) 37 week old WT male (**b**) 51 week old *cap2*^*loxp*^/*cap2*^*loxp*^ male ((**c**), 37 week old and (**d**), 69 week old) *cap2*^−^/*cap2*^−^ males; (**e**) 72 week old *cap2*^*+*^/*cap2*^−^ female (**f**) 54 week old *cap2*^−^/*cap2*^−^ female; (**g**) ANP staining of WT male (**h**) ANP staining of the *cap2*^−^/*cap2*^−^ male from panel d. Size bar 25 μΜ. Areas staining blue indicate fibrosis. (**i**) Expression of CARP in *cap2*^−^/*cap2*^−^ and cardiomyocyte specific knockout mice.

We centered our analysis on the hearts of *cap2*^−^/*cap2*^−^ mice because the male *cap2*^−^/*cap2*^−^ mice were prone to sudden death without any other noticeable signs of distress and CAP2 is highly expressed in heart muscle. When we analyzed the hearts from 7 *cap2*^−^/*cap2*^−^ (3 males and 4 females), 2 *cap2*^−^/*cap2*^*+*^ and 6 *cap2*^*+*^/*cap2*^*+*^ mice by histology, trichrome staining revealed a modest increase in right and left ventricular chamber size with abundant interstitial fibrosis separating and replacing the cardiomyocytes throughout the myocardium of one *cap2*^−^/*cap2*^−^ male (blue staining areas) and a more modest interstitial fibrosis in a second male. None of the 4 female *cap2*^−^/*cap2*^−^ mice showed significant differences from controls (Fig. 4). The male in Fig. 4d, which had extensive myocardial fibrosis, also had increased ANP staining in the left ventricle, which has been linked to the severity of clinical heart failure in humans (Fig. 4g,h)^36^, although ANP was not detected in the other hearts (not shown). In addition, cardiac ankyrin repeat protein (CARP), a transcriptional regulator that is often up-regulated with heart failure, was also elevated, more often in mutant male than female mice. A strong signal was observed in three of 3 males, but only in one of 3 females (Fig. 4i).

**Figure 5:**
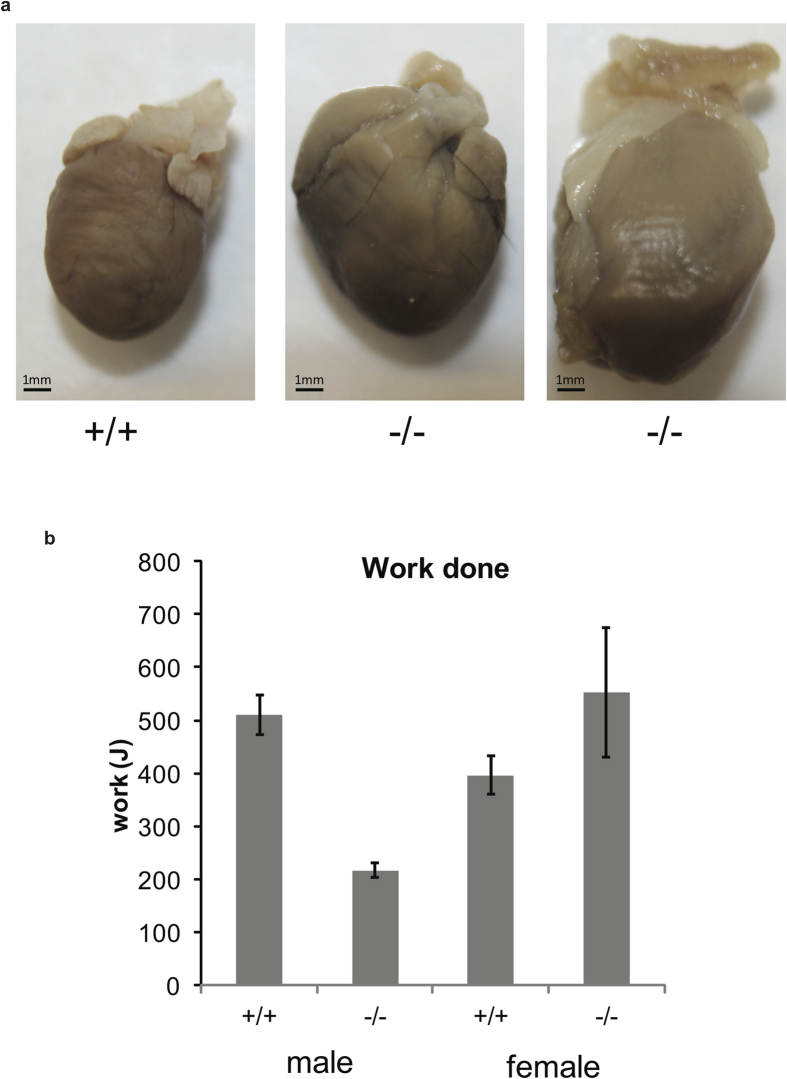
Cardiovascular phenotypes. (**a**) Examples of mouse hearts (**b**) Endurance on a treadmill; work done at 11 weeks of age. Error bars ± S.E.M. Photos by GV and Michelle Skettini.

The hearts from older *cap2*^−^/*cap2*^−^ males showed signs of mild chamber dilatation (Fig. 5a). In addition, as a measure of endurance, we subjected mice to treadmill exercise tests. The male *cap2*^−^/*cap2*^−^ mice traveled about half as far and did less work than sex-matched wild-type controls, but female *cap2*^−^/*cap2*^−^ mice performed as well as the controls in the treadmill test (Fig. 5b).

**Figure 6:**
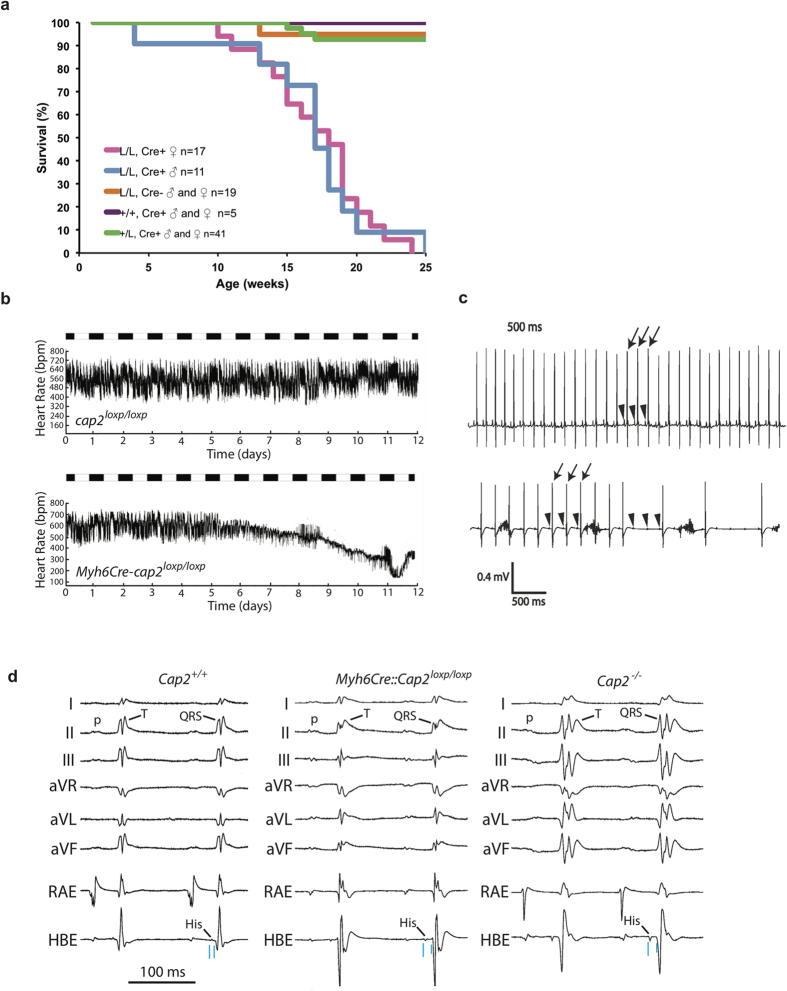
Cardiac conduction disease and sudden cardiac death by heart block in cardiomyocyte-specific knockouts. (**a**) survival of cardiomyocyte-specific knockout mice. (**b**) Left, plot of average heart rate in control littermate mice (top) cardiomyocyte-specific CAP2-null (bottom) over the 12 days prior to sudden death of the CAP2-null mice. Note that about 5 days prior to death, the average heart rate begins to drop in the CAP2-null mice but remains stable in the control mice. (**c**) Loss of CAP2 leads to heart block and sudden death. Representative ECG recordings at the time of death from ambulatory mice using a telemetry recording system. The top trace is from a littermate control mouse recorded at exactly the same time while the bottom trace is from a cardiomyocyte-specific CAP2-null mouse at the time of its death. Note that at this time the initial p-wave to p-wave intervals (arrowheads) is longer in the CAP2-null mouse, as is the QRS-complex to QRS-complex intervals (arrows). The rhythm then degenerates to high-grade AV block in the CAP2-null mouse (several consecutive p-waves with no conducted QRS-complexes) while it remains normal in the control mouse. (**d**) Representative surface ECG and intracardiac tracings from CAP2-null mice. Shown are surface ECGs leads (I through aVF), along with the intracardiac electrogram from the RA (RAE) and His bundle region (HBE) from an 81 week old female, *cap2*^*+*^/*cap2*^*+*^mouse (lefthand), a 19 week old male Myh6Cre-cap2^loxp^/cap2^loxp^ mouse (middle), and an 81 week old female *cap2*^−^/*cap2*^−^mouse (righthand). Note the p-wave morphology (P), QRS-complex (QRS), PR-interval and T-wave morphology (T) are similar between the *cap2*^*+*^/*cap2*^*+*^ and Myh6Cre-cap2^loxp^/cap2^loxp^ mice, but these are both significantly different from the ECG of the *cap2*^−^/*cap2*^−^ mouse. In addition, the HV-interval is prolonged in both CAP2 mutant mice, compared to the control mouse, and these intervals are highlighted by the light blue vertical line at the bottom of each HBE.

Next, because histological analysis suggested the hearts of *cap2*^−^/*cap2*^−^ mice were abnormal, we used transthoracic echocardiography to study heart function in mice as they age. In female *cap2*^−^/*cap2*^−^ mice ranging from 9 to 109 weeks we found that left ventricular chamber size, function and wall thickness were unchanged compared to age-matched control mice (Supplementary Table S2). In male *cap2*^−^/*cap2*^−^ mice, on the other hand, while at 10-weeks and 21-weeks of age, we did not observe changes in left ventricular chamber size, function and wall thickness (Supplementary Table S1), we did find changes in older males. At 55-weeks of age, the *cap2*^−^/*cap2*^−^ males showed increased left ventricular diameter during systole (LVIDs/BW) and reduced LV ejection fraction (Supplementary Tables S1& S2). We also noted several anomalies on the surface ECGs including a prolonged QT interval and increased QRS duration in both male and female *cap2*^−^/*cap2*^−^ mice as they aged. However, the rate corrected QT-intervals (QTm) were not significantly different for both male and female *cap2*^−^/*cap2*^−^ mice compared to control mice (Supplementary Tables S3& S4; Fig. 6). Interestingly, all ECG parameters were normal in male and female *cap2*^−^/*cap2*^−^ mice that were ~30-weeks old and younger. Taken together these data suggest that CAP2 mutant mice initially have normal cardiac function and conduction that degenerates later in life with ventricular conduction system disease and mild, dilated cardiomyopathy that is more pronounced in males versus females.

### Sudden cardiac death by heart block

To generate conditional knockouts, we first excised the insert by crossing mice with actin-flp to generate *cap2*^*loxp*^/*cap2*^*loxp*^. As expected, a western blot demonstrated that *cap2*^*loxp*^/*cap2*^*loxp*^ mice expressed full-length CAP2 (Fig. 1c). Males were born in higher numbers, did not develop eye infections, and no longer died suddenly and isolated *cap2*^*loxp*^/*cap2*^*loxp*^ hearts appeared normal.

To determine which phenotypes of the CAP2 mice are caused by loss of CAP2 in the heart, we generated cardiomyocyte-specific CAP2-null mice by crossing *cap2*^*loxp*^/*cap2*^*loxp*^ mice with *Myh6Cre* mice (B6.FVB-Tg(*Myh6Cre*)2182Mds/J stock number 011038, Jackson Labs), which express Cre-recombinase in cardiomyocytes but not in other myocytes or cell lineages. These progeny were crossed to *cap2*^*loxp*^/*cap2*^*loxp*^ mice or to *cap2*^*+*^/*cap2*^*loxp*^ mice. We were unable to detect CAP2 expression in the *Myh6Cre*-*cap2*^*loxp*^/*cap2*^*loxp*^ hearts on western blots, but could still detect CAP2 in skeletal muscle (Fig. 1c). Histological analysis of hearts from cardiomyocyte-specific CAP2-null mice using trichrome staining showed no evidence of increased interstitial fibrosis, change in gross morphology or cardiac chamber size at ~15 weeks of age (not shown). Interestingly, all of the cardiomyocyte-specific CAP2-null mice were healthy with no apparent discomfort, but died suddenly between 12 and 24 weeks of age (n = 28). Because heart specific expression of Cre by some promoters can cause cardiomyopathy in the absence of floxed test genes^37^,^38^, we performed survival controls on *Myh6Cre*-*cap2*^*+*^/*cap2*^*loxp*^ (n = 41) mice and also on *Myh6Cre*-*cap2*^*+*^/*cap2*^*+*^ (n = 5) mice. Both of these two genotypes had much higher survival rates than *Myh6Cre*-*cap2*^*loxp*^/*cap2*^*loxp*^ mice, with only 3 dying by 25 weeks (Fig. 6a). Thus, we did not see any effects of the *Myh6Cre* transgene in our system. As a result, we used these mice interchangeably as controls for Echo and ECG.

Unlike total body knockouts, both male and female cardiomyocyte-specific CAP2-null mice died at about the same age so there was no longer a sex bias in viability (Fig. 6a). We found that 20 *Myh6Cre*-*cap2*^*loxp*^/*cap2*^*loxp*^ males vs. 24 females survived to weaning, which is similar to the number of cap2^loxp^/cap2^loxp^ (L/L, Cre-) mice that survived to weaning. Thus, the decrease in viability of these mice appears to be caused by the loss of CAP2 in cardiomyocytes, while the male bias in sudden death appears to be due to the loss of CAP2 in tissues outside of cardiomyocytes.

To determine the cause of death in the cardiomyocyte-specific CAP2-null mice, we performed ambulatory ECG monitoring and invasive electrophysiology studies in these animals. For these studies we monitored six (4 cardiomyocyte-specific CAP2-null and 2 control) animals using ECG telemetry and found that all 4 cardiomyocyte-specific CAP2-null mice first developed sinus bradycardia (Fig. 6b) and then died a few days later from complete atrioventricular block (Fig. 6c). In contrast, none of the control mice showed any evidence of conduction defects or arrhythmias. Interestingly, when we compared the ambulatory surface ECG intervals between cardiomyocyte-specific CAP2-null and control mice we did not see any differences in the ECG intervals within the first few weeks after implanting the ECG monitor (Table 2). However, when we compared the ambulatory surface ECG intervals between these two groups at the same time points corresponding to 1 hour prior to the death of the CAP2-null mice, we found that the RR-intervals and QRS-intervals were longer in the CAP2-null mice versus the control mice (Table 2). Furthermore, when we performed invasive electrophysiological studies in 12–20 week-old cardiomyocyte-specific CAP2-null and age-matched control mice, we saw a significant increase in the HV-interval and AV Wenckebach cycle length in cardiomyocyte-specific CAP2-null mice versus control mice (Table 3). In addition, we performed invasive EP studies in two older *cap2*^−^/*cap2*^−^ and control mice and found the HV-intervals (14 vs. 10) and AV Wenckebach cycle lengths (112 vs. 96) were also longer in these animals (Fig. 6c,d). On the other hand, we did not see significant differences in the surface ECG intervals, sinus node recovery times or arrhythmia induction between the two groups, although QRS-duration was approaching significance for being wider in cardiomyocyte-specific CAP2-null mice (Supplementary Table S5) and became significant if the data from males and females were pooled. Furthermore, transthoracic echocardiographic analysis of cardiomyocyte-specific CAP2-null mice aged 12–20 weeks showed no difference in left ventricular wall thickness, chamber size or function in male or female mice compared to control mice (Supplementary Table S6). These findings suggest that CAP2 is necessary for proper function of the postnatal CCS and, if it is misexpressed or dysregulated in cardiomyocytes, may contribute to sudden death in the absence of significant structural heart disease.

## Discussion

CAP2 knockout mice are distinguished by (1) short stature, (2) dilated cardiomyopathy (3) sudden cardiac death caused by heart block and (4) microphthalmia. Just after birth, *cap2*^−^/*cap2*^−^ hearts are normal in size. As early as ~12 weeks, sudden death occurs, but cardiac enlargement and dilatation develop at about 6 months of age, somewhat sooner for males. Fibrosis, when observed, is not present until about 12 months. Surprisingly, there is a strong sex bias, with most of the phenotypes more commonly seen in males than females. The sex bias is lost in cardiomyocyte-specific knockouts because males and females are born in equal numbers and both males and females die at about the same age from heart block. Since the primary function of all CAP homologs is actin monomer binding and CAP mutants in yeast, Drosophila or mammalian cells (knockdown) disrupt the actin cytoskeleton, it is likely that the phenotypes in CAP2 knockouts are caused by dysfunction of the cytoskeleton, although at the ultra-structure level, sarcomeres show no gross abnormalities near the time of death.

Peche *et al.* reported that CAP2 knockout mice developed DCM and speculated that KO males died from sudden cardiac death due to ventricular arrhythmias^39^. Although we also observe mild DCM in *cap2*^−^/*cap2*^−^ mice, we conclude that the basis of the sudden cardiac death is CCD with heart block. In *cap2*^−^/*cap2*^−^ males, there is a critical period at 5–8 weeks of age, during which most mice die. It is the survivors that slowly develop DCM, but compared to other animal models, the DCM is relatively mild enabling the mice, especially females, to survive about as long as wild-type mice. For example, MLP-deficient mice develop DCM with a higher penetrance, and at much earlier ages than CAP2 KO mice^40^. On the other hand, all of the *cap2*^−^/*cap2*^−^ mice develop cardiac conduction dysfunction on ECG and by invasive analysis and, in cardiomyocyte-specific knockouts, all of the mice die from complete heart block before there is significant DCM. In tracing the source of the cardiac conduction problems, we found *cap2*^−^/*cap2*^−^ mice have evidence of compromised function in both the suprahisian conduction system (prolonged AV Wenckebach cycle length) and the infranodal conduction system (prolonged HV-intervals). Although further investigation is needed to determine which anatomic CCS structures are functionally affected at the time of death in these mice, there is little doubt that loss of CAP2 contributes to complete heart block and sudden death in this model.

Patients with a cardiomyopathy and evidence of cardiac conduction system disease are at higher risk for sudden cardiac death^1^. Most cases of sudden cardiac death are the result of unrecognized cardiac disease prior to the manifestation of electrocardiographic changes or symptoms^1^,^5^,^6^. Interestingly, *cap2*^−^/*cap2*^−^ mice phenocopy many of the same features of one such population of patients, those with mutations in lamin A/C. Patients with lamin A/C mutations initially have no overt cardiac disease or symptoms, but go on to develop AV conduction defects, sinus bradycardia and atrial arrhythmias before their fourth decade of life, followed by the development of dilated cardiomyopathy and sudden cardiac death in their fourth to fifth decades of life^5^. LMNA^N195K^ is a mutation in nuclear lamin A/C found in some families that causes cardiac conduction disease and mild DCM. A LMNA^N195K^ mouse model shows remarkable similarities to CAP2 knockouts. LMNA^N195K^ mice die at about 12 weeks of age of heart block after ~3 days of severe bradycardia but show only a mild DCM^41^. LMNA mutant males also have reduced survival compared to females^41^,^42^. The CAP2 null mice also develop cardiac conduction disease and heart block prior to the onset of DCM, although they do not develop ventricular arrhythmias. The similarities in these phenotypes suggest there may be a pathway common to CAP2 and lamin A/C in the heart.

Most mutations that cause DCM are in cytoskeletal proteins that constitute the sarcomere, but several phenotypic features of the CAP2 null mice suggest that it is an atypical DCM gene and resembles those more commonly found in patients with mutations in genes encoding ion channels or gap junction proteins^17^,^43^. First, the male bias of the cardiac phenotypes is rarely seen in mouse models of DCM. However, there is a male bias in several transgenic mouse models involving channel proteins^44^. Second, CAP2 cardiomyocyte-specific knockout mice develop bradycardia and heart block, as do mice that have mutations in the pacemaker channel Hcn4^45^ or the gap junction protein connexin40 (Cx40)^46^. There are a few examples of mutations in cytoskeletal genes causing cardiac conduction disease and heart block. These include overexpression of the small GTPase Rho and, as discussed above, expression of the mutant lamin A, LMNA^N195K ^41,47. Rho expression, like loss of CAP proteins, stimulates the formation of actin filaments. While LMNA has been widely studied as a nuclear envelope protein, it was recently shown to regulate actin dynamics^4^. All three of these proteins have the ability to regulate the actin cytoskeleton, but are not exclusively sarcomere proteins. CAP2 may act independently of Rho and LMNA or be the functional link between the cytoskeleton and the cardiac conduction system.

An unexpected observation is the strong sex bias in survival, with only ~30% of weaned knockout males surviving past 12 weeks. The sex bias appears to reflect a difference in the onset of cardiac disease as CARP was induced more frequently in males than in females. There is male bias in many congenital heart diseases including patients with lamin A/C mutations^48^,^49^ and women with heart failure live significantly longer than men. Women with cardiac conduction disease also live longer, even after pacemakers are implanted. Thus the sex-bias in CAP2 knockout mice may be a useful model system for studying sex-differences in the progression of cardiomyopathy and cardiac conduction disease.

A difference between the cardiomyocyte-specific knockouts and the whole body knockouts is that all cardiomyocyte-specific knockout mice die prematurely whereas only ~70% of the male whole body knockouts die suddenly by 12 weeks of age, although the survivors later develop mild cardiomyopathy later in life. However, the loss of CAP2 likely causes postnatal mortality early in life in the whole body knockout before they can be genotyped, so the whole body KO survival curves are biased toward suggesting knockouts have higher survival rates. A likely explanation for the differences observed in survival between cardiomyocyte-specific KO and whole body KO mice is that the loss of CAP2 in tissues other than the heart affects the secretion of factors, such as sex hormones, that can influence the progression of cardiac conduction disease. In this regard, it is interesting that CAP2 is more highly expressed in the testis than any organ except the brain, heart and skeletal muscle.

The high expression levels of CAP2 in skeletal muscle may reflect a non-redundant role for CAP2 in this tissue. Although we do not observe obvious muscle abnormalities, mutation of the related cytoskeletal gene, cofilin-2, causes muscle degeneration by day 7 and the animals die at a few weeks of age because they are unable to suckle milk from their mothers^50^. We have not noted any obvious problems nursing in *cap2*^−^/*cap2*^−^ mice, but it is possible that the short stature and slow growth may be caused by a reduced ability to nurse. We can conclude that the short stature is caused by loss of CAP2 in tissues other than the heart because cardiomyocyte-specific knockouts are normal in size and weight.

CAP2 knockouts are prone to eye infections, which are likely caused by microphthalmia. C57BL/6, and other strains of inbred mice are prone to microphthalmia, which occurs with a frequency of 4% to 10% and is predominantly seen in the right eyes^51^. The microphthalmia causes poor irrigation, preventing tears from flushing debris, which then seed infections. The underlying problem is improper formation of the optic cup. There is also a sex bias in other models of microphthalmia; however, in other models, microphthalmia is seen predominantly in females, while CAP2 knockouts predominantly show microphthalmia in males. Mice with mutations in a cofilin homolog develop a cloudy cornea and eventually lose their eyesight^52^. We do not observe any cloudiness in the corneas of CAP2 KO mice, suggesting a different mechanism. Further, both electroretinograms and pupillometry results demonstrated normal light sensitivity, which would not have been apparent if there were opacities in the light pathway (as well as intact CNS pupillary light reflex pathways). A more likely explanation for the eye phenotypes is that the cytoskeleton regulates the expression of key developmental genes, such as hedgehog and hippo, as seen in *Drosophila*^30^,^31^.

The phenotypes of short stature, cardiac defects, eye abnormalities, and developmental delays are similar to those observed in patients with 6p22 syndrome^13^. Relatively little is known about the other genes in the region, although ATXN1 is likely to contribute to the cognitive phenotypes because ATXN1 knockout mice^15^ show learning and memory deficits but not heart or eye problems^53^. While 6p22 syndrome patients have relatively large deletions, one study narrowed the overlapping region to 12 genes and another to four genes; CAP2 was in the overlap region in both studies^12^,^15^. Other studies localized a gene for noncompaction cardiomyopathy through three generations in two families to 6p24.3–21.2 54. In addition, genome-wide association studies (GWAS) mapped single nucleotide polymorphisms (SNPs) near CAP2 for hypertension^7^, widened QRS complexes^8^, left ventricular hypertrophy^9^, coronary artery disease^10^, sudden cardiac death^1^, and height^11^. Although the SNPs are not in exons, they may affect CAP2 expression and our data suggest that heterozygous loss of CAP2, both whole body and heart specific, is a risk factor for sudden cardiac death.

## Methods

### Generation of CAP2 knockout mice

We obtained three different ES clones (C08, E05 and D07) European Conditional Mouse Consortium (EUCOMM). Two clones passed our quality control PCR tests (C08, E05) and were injected into Balb/c embryos (Charles River Laboratories, Wilmington, MA), which were then transferred to pseudopregnant CD1 females (Charles River Laboratories); chimeric offspring were identified by the presence of black hair. Chimeric males were mated to C57BL/6J females to obtain ES cell-derived offspring, which were analyzed by PCR of toe DNA to identify the CAP2 heterozygote mice. The *cap2*^−^ construct inserts sequences that disrupt the CAP2 gene along with FRT sites flanking the insert and loxP sites flanking exon 3. Crossing *cap2*^−^ mice to actin-FLP mice (B6.Cg-Tg(ACTFLPe)9205Dym/J; stock number 005703, Jackson Labs) created *cap2*^*loxp*^ mice, which deleted most of the insert restoring CAP2 expression but leaving the two loxP sites flanking exon 3. Crossing the *cap2*^*loxp*^ mice to Myh6-Cre mice (B6.FVB-Tg(*Myh6Cre*)2182Mds/J; stock number 011038, Jackson Labs) created *cap2*^*loxp*^/*cap2*^*loxp*^Myh6-Cre, which are heart specific deletions of CAP2. Figure 1a shows the three transgenic genotypes, *cap2*^−^/*cap2*^−^, *cap2*^*loxp*^*/ cap2*^*loxp*^ and *cap2*^*loxp*^/*cap2*^*loxp*^Myh6-Cre.

### Genotyping

The following primers were used for CAP2 genotyping: Cap2–2lox RP (5′-ACC CCA CAT TTA CGA TGG CTC CGG-3′), Cap2-LAR3 (5′-CAA CGG GTT CTT CTG TTA GTC C-3′), and Cap2–5′ arm N (5′-TAC CTG GAA GAG CTA CAG AGG-3′). Typically, a CAP2 genotyping reaction utilized all three primers. To confirm the presence or absence of the WT band in cases of ambiguity, only two of the primers were used (Cap2–2lox RP and Cap2–5′ arm N). To detect the presence of Myh6-Cre, the following two primers were used: cre-F (5′-TGC CAC GAC CAA GTG ACA GC-3′) and cre-R (5′-CCA GGT TAC GGA TAT AGT TCA TG-3′). Examples both genotyping reactions are shown in Fig. 1a.

### Histology and Immunohistochemistry

Detailed protocols used for histological and immunohistochemical staining have been previously described^55^. For Masson’s trichrome staining the hearts were fixed in 4% paraformaldehyde, dehydrated through an ethanol series, embedded in paraffin, sectioned and then stained. Immunohistochemistry was also performed on fixed, paraffin embedded sections with rabbit anti-ANP antibodies (sc-18811, Santa Cruz Biotechnology). Primary antibodies were recognized by Alexa488 goat anti-rabbit antibodies (Jackson ImmunoResearch Laboratories).

### Western blots

Whole cell lysates were prepared from heart and muscle and 25–30 μg of protein was resolved by SDS-PAGE (4–12% gel, Invitrogen). The proteins were transferred to PVDF (Invitrogen) membranes and probed with an anti-CAP2 antibody (Sigma, C-7246, 1:1000) or an anti-CARP antibody (sc-30181 Santa Cruz Biotechnology). Antibodies against β-actin (Santa Cruz, SC-1615,1:1000), or GAPDH (sc-25778 Santa Cruz Biotechnology) were used as loading controls. CAP2 is ~56 kDa, but some gels were run under non-reducing conditions where CAP2 ran as a dimer of ~98 kDa. Immunoreactivity was visualized by use of ECL (manufacturer’s protocol).

### Transthoracic Echocardiography

Mice were anesthetized by inhalation of 4% isoflurane in a glass chamber and then maintained during procedures in 1% to 1.5% isoflurane via nose cones. Animals were imaged on a heated platform while monitoring body temperature and a single-lead ECG through the platform. Echocardiography was performed using a Vevo 770 (VisualSonics) with a linear 30-MHz probe (RMV 707B). M-mode images were obtained for measurement of wall thickness, chamber dimension and fractional shortening.

### Electrophysiology studies

Surface ECG and invasive mouse electrophysiology studies were performed as described^55^,^56^. Each mouse was anesthetized with 0.75–1.5% isoflourane plus 60% oxygen, and multi-lead ECGs obtained using 26-gauge subcutaneous electrodes. Core body temperature was maintained with an infrared heating lamp at 33–34 °C and monitored with a rectal probe. A jugular vein cutdown was performed and an octapolar 1.0-French electrode catheter (EP800, Millar Instruments Inc. Houston, TX) placed in the right atrium and ventricle under electrogram guidance to confirm catheter position. A programmed digital stimulator (DTU-215A, Bloom Associates Ltd., Reading, PA) was used to deliver electrical impulses at approximately twice the diastolic threshold. Surface ECG and intracardiac electrograms were simultaneously displayed on a multichannel oscilloscope recorder (Bard Electrophysiology, Inc. Lowell, MA) at a digitization rate of 2 kHz and stored on optical media for offline analysis. ECG channels were filtered from 0.5–250 Hz and intracardiac electrograms were filtered from 5–400 Hz. ECG intervals were measured by two independent observers blinded to the animal’s genotype. We considered an arrhythmic episode to be the induction of three or more consecutive ectopic beats following the last extrastimuli of a drive train.

### Ambulatory ECG recordings

Ambulatory ECG recordings were obtained by aseptic, subcutaneous implantation of a 1.1-g wireless radiofrequency telemetry device (ETA-F10; Data Sciences International, St. Paul, MN) configured to record a signal analogous to ECG lead I as previously described^55^. Following a 7 day recovery period, continuous ECG recordings were obtained for at least 24 hours from cardiac-specific CAP2 knockout and control littermate mice in separate cages overlying a receiver. ECG intervals were measured by two independent observers blinded to the genotypes using digital calipers in the LabChart 5.0 analysis suite (ADInstruments, Inc., Colorado Springs, CO).

### Ethics approval

All animal studies were reviewed and approved by the University of Pennsylvania IACUC and studies were carried out in accordance with the approved guidelines..

## Additional Information

**How to cite this article**: Field, J. *et al.* CAP2 in cardiac conduction, sudden cardiac death and eye development. *Sci. Rep.* **5**, 17256; doi: 10.1038/srep17256 (2015).

## Supplementary Material

Supplementary Information

## Figures and Tables

**Table 1:** Genotypes of pups from *cap2*^−^/*cap2*^*+*^ × *cap2*^−^/ *cap2*^*+*^crosses.

Gender	Genotype	Number of mice	Percentage
Male	*cap2*^+^*/cap2*^+^	14	25%
	*cap2*^−^/cap2^+^	39	69.7%
	*cap2*^−^/*cap2*^−^	3	5.3%
	**Total**	56	
Female	*cap2*^*+*^*/cap2*^*+*^	14	23.7%
	*cap2*^−^*/cap2*^*+*^	35	59.4%
	*cap2*^−^/*cap2*^−^	10	16.9%
	**Total**	59	

**Table 2:** Ambulatory ECG Intervals.

	14 Days Post-Monitor Implant		1 Hour Prior to Death
	Control (n = 2)	Myh6Cre-cap2^loxp^/cap2^loxp^ (n = 4)	Myh6Cre-cap2^loxp^/cap2^loxp^ (n = 4)
Age (days)	106	104 ± 10	131 ± 17
RR (ms)	138	132 ± 22.8	567 ± 19.2^A^
HR (bpm)	435	455 ± 31.6	106 ± 25.4^A^
PR (ms)	35.2	36.8 ± 3.4	35.2 ± 3.9
P-wave (ms)	17.2	18.2 ± 2.4	18.5 ± 3.1
QRS (ms)	9.8	10.0 ± 1.0	13.8 ± 1.5^A^
QT (ms)	23.8	24.6 ± 3.4	29.4 ± 3.8
QTm (ms)	19.6	19.4 ± 1.6	21.8 ± 1.5

^A^p < 0.05 for Myh6Cre-cap2^loxp^/cap2^loxp^ 14 days post-implant versus Myh6Cre-cap2^loxp^/cap2^loxp^ 1 hour before death. RR = R-wave to R-wave interval; HR = Heart rate; PR = PR-interval duration; QRS = QRS-complex width; QT = QT-interval duration; QT_m_ = Heart-rate corrected QT-interval duration.

**Table 3:** Baseline invasive electrophysiology intervals.

	Control (n = 5)	Myh6Cre-cap2^loxp^/cap2^loxp^ (n = 5)
AH (ms)	32.5 ± 5.2	33.1 ± 4.7
H_d_ (ms)	3.8 ± 1.0	3.9 ± 0.9
HV (ms)	10.2 ± 1.6	13.7 ± 1.8^A^
AVI (ms)	42.6 ± 5.9	46.6 ± 5.6
SNRT120 (ms)	211 ± 58.0	207 ± 54.5
SNRT100 (ms)	226 ± 68.4	230 ± 55.6
AVWCL (ms)	94.5 ± 7.2	106 ± 7.6^A^
AVERP120(ms)	69.9 ± 9.2	73.8 ± 8.5
AERP120 (ms)	42.4 ± 5.5	44.6±5.9
AERP100 (ms)	40.8 ± 5.2	42.4±6.42
VERP120 (ms)	36.9 ± 8.4	37.5 ± 8.9
VERP100 (ms)	34.6 ± 9.8	35.7.0 ± 9.6
Episodes AT	5	7
Episodes VT	3	2
AT duration (ms)	278 ± 48.1	312 ± 56.0
VT duration (ms)	342 ± 50.8	289 ± 52.6
Age (d)	89.4 ± 16.2	90.7 ± 15.8
Weight (g)	24.6 ± 4.4	25.0 ± 4.8

^A^p < 0.05; AH = Atrio-hisian interval; H_d_ = His-duration; HV = Hisioventricular interval; AVI = Atrioventricular interval; SNRT_120_ = Sinus node recovery time at drive train of 120ms; SNRT_100_ = Sinus node recovery time at drive train of 100 ms; AVWCL= AV Wenckebach block cycle length; AVERP_120_ = Atrioventricular ERP at drive train of 120ms; AERP_120_ = Atrial ERP at drive train of 120 ms; AERP_100_ = Atrial ERP at drive train of 100 ms; VERP_120_ = Ventricular ERP at drive train of 120ms; VERP_100_ = Ventricular ERP at drive train of 100 ms; AT = atrial tachycardia; VT = ventricular tachycardia; Mean AT CL = mean atrial tachycardia cycle length. Data are presented as the mean ± standard deviation and tests of statistical difference were computed using one-way Student’s t-test all intracardiac parameters, animal weights and ages. The numbers of arrhythmic episodes were assumed to have a Poisson distribution and the Kolmogorov-Smirnov test was used to assess statistical significance between groups.
