# Erratum: CAP2 in cardiac conduction, sudden cardiac death and eye development

**DOI:** 10.1038/srep26640

**Published:** 2016-05-31

**Authors:** Jeffrey Field, Diana Z. Ye, Manasi Shinde, Fang Liu, Kurt J. Schillinger, MinMin Lu, Tao Wang, Michelle Skettini, Yao Xiong, Angela K. Brice, Daniel C. Chung, Vickas V. Patel

Scientific Reports 5: Article number: 1725610.1038/srep17256; published online: 11302015; updated: 05312016

This Article contains typographical errors in the legend of Supplementary Figure S1,

“Physiology phenotyping (a and b) DEXA analysis of mice. (c) Grip strength of mice (d) Glucose tolerance test. Error bars ± S.E.M.”

should read:

“Physiology phenotyping (a and c) DEXA analysis of mice. (b) Grip strength of mice (d) Glucose tolerance test. Error bars ± S.E.M.”

