# Supplementary material for: CAP2 in cardiac conduction, sudden cardiac death and eye development
Source: Sci Rep. 2015 Nov 30;5:17256. doi: 10.1038/srep17256 (PMC4663486; doi:10.1038/srep17256)
Supplement: Supplementary Information [file srep17256-s1.pdf]

CAP2 in cardiac conduction, sudden cardiac death and eye development

Jeffrey Field PhD, Diana Z. Ye, PhD, Manasi Shinde, Fang Liu, Kurt J. Schillinger, MD,PhD MinMin Lu, Tao Wang, Michelle Skettini, Yao Xiong, Angela K. Brice, DVM, PhD, Daniel C. Chung, DO and Vickas V. Patel, MD,PhD

Supplementary methods

Quantitative real-time PCR

The heart, muscle and brain were dissected and total RNA extracted in TRIzol (Invitrogen, Carlsbad, CA) according to the manufacturer's protocol. For quantitative real-time PCR, the reaction mixture was prepared using TaqMan Gene Expression Assays and Fast Universal PCR Master Mix (Applied Biosystems, 4352042). Reactions were performed using the Step-one Plus Quantitative PCR System (Applied Biosystems). All reactions were performed in triplicate with reference dye normalization, and median threshold cycle values were used for analysis. The CAP2 primers used were Mm00482645_m1 and the GAPDH Primers were: Mm99999915_g1, (both from Applied Biosystems).

Whole Body Dual X-Ray Absorptiometry (DEXA)

DEXA was performed to determine the whole body composition of mice. This test measured the bone mineral content, lean muscle and fat content. Eleven-week old male mice were injected intraperitoneally with Ketamine/Xylazine (100 mg/kg) and dual energy x-ray absorptiometry was performed (DEXA; Lunar PIXImus2; General Electric Medical systems, Madison, WI) to determine their body composition. Following the 3 minute scan, mice were placed under heat lamp and monitored continuously until recovery from anesthesia.

Grip strength test

Grip strength test was performed to determine the skeletal muscle strength of the CAP2 KO mice and controls. Forelimb grip strength was tested using a Gripmeter (TSE systems). Eleven-week old male mice were held by the tail and allowed to grasp a horizontal bar connected to a force detector. Grip strength was measured instantaneously for 3 trials.

Treadmill exercise and energy expenditure tests

Treadmill exercise and energy expenditure tests were performed on a belt that can be adjusted for speed and slope. The test chamber also has a stimulus device consisting of a metal shock grid attached to the rear of the belt. In tests, stimulus shocks ranged from 1.8 milli-units to 3.6 milli-units and the belts were run for 30 minutes at speeds of 10, 15, 20, 25, 30 meters per at inclines ranging from 10 to 15%. If the mice stopped running and did not complete the 30 minutes at a given speed, they were not tested at faster speeds. The O_2 consumption and CO_2 generation were measured and respiratory exchange ratio (RER) was calculated. The O_2 consumption was calculated as the difference between the input oxygen flow and the output oxygen flow. The RER is calculated as the ratio between the CO_2 production and the O_2 consumption.

Electroretinograms (ERGs)

Dark-adapted (overnight) animals were anesthetized with an intramuscular injection of a mixture of ketamine HCl (65 mg/kg) and xylazine (5 mg/kg) and pupils were dilated with tropicamide(1%) and phenylephrine (2.5%). Full-field bilateral ERGs were recorded using a custom-built ganzfeld, a computer-based system (EPIC-XL; LKCTechnologies, Inc, Gaithersburg, MD, USA), and specially made contact lens electrodes (Hansen Ophthalmics, Iowa City, IA, USA). Medium- and high-energy (10 As and 1ms duration, respectively) flash stimulators (with unattenuated maximal white flash luminances of 0.8 and 3.6 log scot-cd s m2, respectively) were used. Neutral density (Wratten 96; Kodak, Rochester, NY, USA) and blue (Wratten 47A) filters served to attenuate and spectrally shape the stimuli. First, dark-adapted ERGs were obtained with increasing intensities (4.2 to 0.1 log scot-cd sm2) of blue flashes.

Next, dark-adapted ERG photoresponses were evoked with two flash intensities (blue 2.2 and white 3.6 log scot-cd sm2).

Pupillometry

Measures of the amplitude of the pupillary light reflex were performed in representative affected and unaffected animals without sedation. A NeurOptics A-1000 Pupillometer (NeurOptics Inc., Irvine, CA, USA) was used to measure changes in pupillary diameter of eyes of the animals following exposure to five 0.1-s flashes of 4.5 μW/cm2 intensity at 10-s intervals. Stimuli were presented to one eye at a time in each test. Amplitudes of the pupillary reflex were compared in affected and unaffected animals.

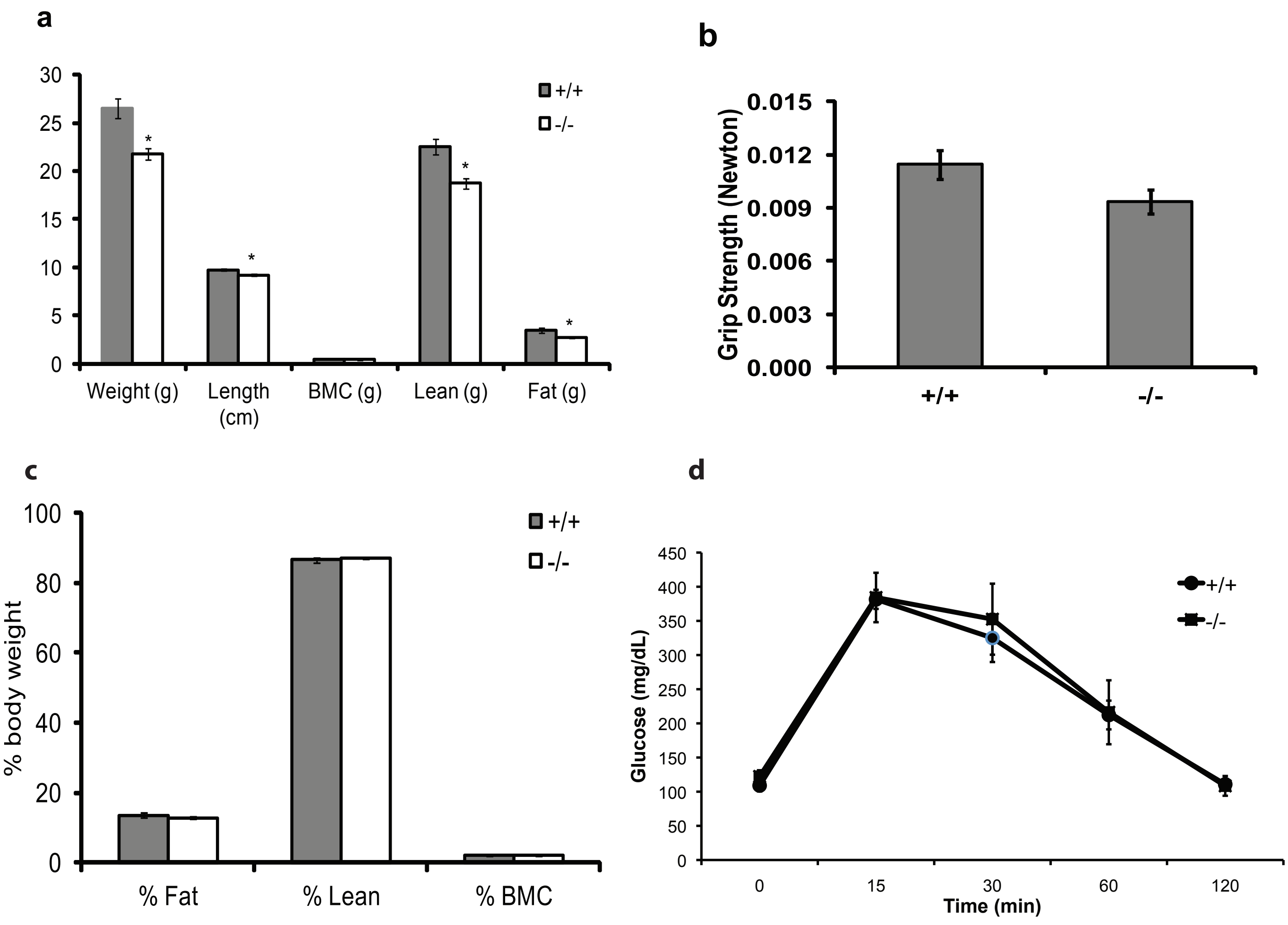

a
30
25
20
15
10
5
0
+/+
-/-
*
*
*
*
Weight (g)
Length (cm)
BMC (g)
Lean (g)
Fat (g)
b
Grip Strength (Newton)
0.015
0.012
0.009
0.006
0.003
0.000
+/+
-/-
c
% body weight
100
80
60
40
20
0
+/+
-/-
% Fat
% Lean
% BMC
d
Glucose (mg/dL)
450
400
350
300
250
200
150
100
50
0
+/+
-/-
0
15
30
60
120
Time (min)

Supplementary Figure S1 Physiology phenotyping **(**a and b) DEXA analysis of mice. (c) Grip strength of mice (d) Glucose tolerance test. Error bars ± S.E.M.

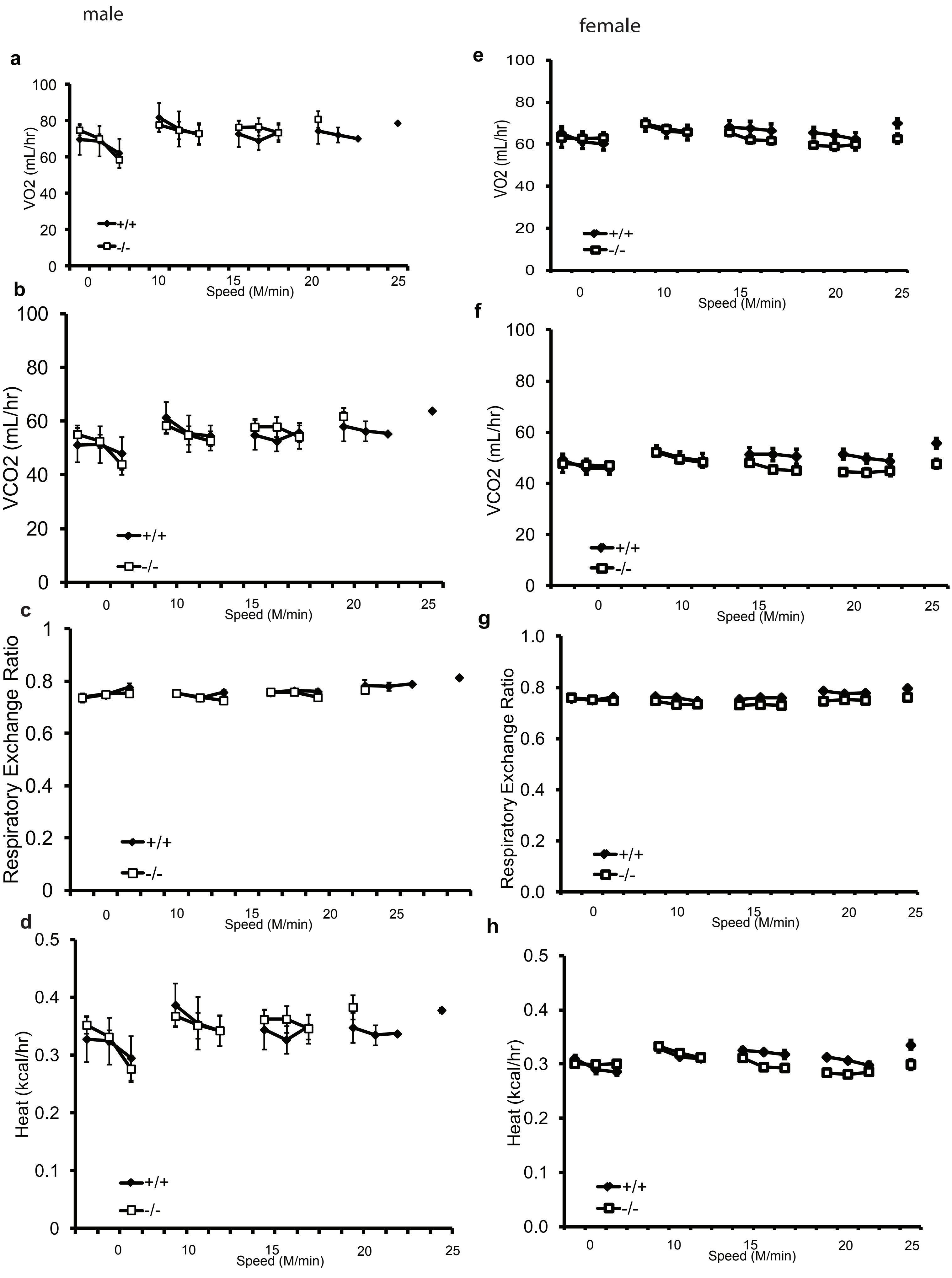

male
female
a
VO2 (mL/hr)
+/+
-/-
Speed (M/min)
b
VCO2 (mL/hr)
+/+
-/-
Speed (M/min)
c
Respiratory Exchange Ratio
+/+
-/-
Speed (M/min)
d
Heat (kcal/hr)
+/+
-/-
Speed (M/min)
e
VO2 (mL/hr)
+/+
-/-
Speed (M/min)
f
VCO2 (mL/hr)
+/+
-/-
Speed (M/min)
g
Respiratory Exchange Ratio
+/+
-/-
Speed (M/min)
h
Heat (kcal/hr)
+/+
-/-
Speed (M/min)

Supplementary Figure S2. Treadmill parameters (a, e) VO_2, (b, f) VCO_2, (c,g) Respiratory exchange ratio (RER), (d, h) Heat generated. Error bars ± S.E.M.

	Males: 10 week-old		Males: 21 week-old		Males: 55 week-old	
Cap2 genotype	+/+	-/-	+/+	-/-	+/+	-/-
IVS (µm)	670 ± 20	690 ± 50	650 ± 40	610 ± 60	670 ± 100	720 ± 100
LVPW (µm)	650 ± 10	690 ± 80	650 ± 30	590 ± 80	680 ± 130	730 ± 140
LVIDd/BW (µm/g)	160 ± 4	200 ± 10	170 ± 10	220 ± 30	190 ± 20	200 ± 10
LVIDs/BW (µm/g)	120 ± 5	150 ± 10	120 ± 10	150 ± 20	130 ± 20	180 ± 20[A]
LVEF (%)	53 ± 3.3	52 ± 6.4	59 ± 4.1	60 ± 4.6	55 ± 5.0	44 ± 4.2[A]

[A]$p < 0.05$ compared to age- and sex-matched genotype. N=3-5 in each group.
IVS = intraventricular septum; LVPW = left ventricular posterior wall; LVIDd = left ventricular internal dimension at diastole; left ventricular internal dimension in systole; left ventricular ejection fraction. BW = body weight.

Supplementary Table S1. Echocardiographic parameters

	Females: 9 week-old		Females: 21 week-old		Females: 81 to 109 week-old	
Cap2 genotype	+/+	-/-	+/+	-/-	+/-	-/-
IVS (µm)	650 ± 40	610 ± 30	620 ± 50	590 ± 10	700 ± 140	690 ± 90
LVPW (µm)	620 ± 70	570 ± 90	600 ± 50	600 ± 30	680 ± 120	690 ± 100
LVIDd/BW (µm/g)	200 ± 10	230 ± 10	200 ± 10	220 ± 20	180 ± 20	210 ± 30
LVIDs/BW (µm/g)	140 ± 10	160 ± 10	130 ± 10	140 ± 20	130 ± 10	150 ± 20
LVEF (%)	58 ± 3.4	56 ± 5.2	68 ± 6.2	67 ± 4.8	50 ± 3.4	49 ± 5.7

[A]$p < 0.05$ compared to age- and sex-matched genotype. N=3-5 in each group.
IVS = intraventricular septum; LVPW = left ventricular posterior wall; LVIDd = left ventricular internal dimension at diastole; left ventricular internal dimension in systole; left ventricular ejection fraction. BW = body weight.

Supplementary Table S2. Echocardiographic parameters

	Males: 12 week-old		Males: 31 week-old		Males: 55 week-old	
Cap2 genotype	+/+	-/-	+/+	-/-	+/+	-/-
P-wave (ms)	19.3±2.2	22.6±2.4	21.8±2.3	21.5±0.7	18.3±2.5	24.3±5.1
RR-interval (ms)	121±28.2	130±22.7	117±3.9	127±1.4	124±7.6	157±43.5
PR-interval (ms)	37.2±2.2	40.1±2.0	40.2±2.6	41.5±2.1	44.3±5.3	49.3±10.4
QRS-duration (ms)	11.2±1.0	13.4±0.9	11.6±1.5	13.5±0.7	14.75±1.0	16.8±0.5[A]
QT-interval (ms)	28.5±1.8	30.7±2.0	33.0±4	32.0±1.4	28.8±2.6	34.8±2.6[A]
QTm-interval (ms)	21.2±1.2	22.5±1.4	23.2±1.6	23.3±2.1	21.5±2.0	23.4±2.2

[A]$p < 0.05$ compared to age- and sex-matched genotype. N=3-5 in each group
QTm=Murine heart-rate corrected QT-interval

Supplementary Table S3. Surface ECG parameters

	Females: 12 week-old		Females: 32 week-old		Females: 41 week-old	
Cap2 genotype	+/+	-/-	+/+	-/-	+/+	-/-
P-wave (ms)	20.4±2.2	21.8±2.0	23.5±1.9	24.8± 1.7	21.0±2.2	22.0±2.6
RR-interval (ms)	119±16.4	137±15.4	128±17.3	137±15.4	118±6.8	120±1.0
PR-interval (ms)	36.5±3.1	37.3±2.7	38.3±2.9	43.0±0.8	39.3±2.9	44.3±1.5
QRS-duration (ms)	10.8±1.2	11.6±1.1	11.3±1.0	12.3±0.5	10.5±0.6	15.0±1.0[A]
QT-interval (ms)	27.7±2.1	29.4±2.0	31.3±0.5	32.8±1.3	27.5±1.6	31.7±1.2[A]
QTm-interval (ms)	22.2±1.4	21.6±1.7	23.4±0.3	21.2±0.9	20.4±1.2	22.1±0.8

[A]$p < 0.05$ compared to age- and sex-matched genotype. N=3-5 in each group.
QTm=Murine heart-rate corrected QT-interval.

Supplementary Table S4. Surface ECG parameters

	Males: 14 week-old		Females: 14 week-old	
Cap2 genotype	Control	*Myh6Cre-cap2^{loxp}/cap2^{loxp}*	Control	*Myh6Cre-cap2^{loxp}/cap2^{loxp}*
P-wave (ms)	19.9±2.0	20.6±2.2	21.5±0.7	21.8±1.3
RR-interval (ms)	128±22.9	179±26.3[A]	126±23.5	183±25.2[A]
PR-interval (ms)	37.9±2.5	37.5±2.1	38.8±2.3	39.1±1.8
QRS-duration (ms)	10.7±0.8	12.4±1.2[B]	10.8±0.8	11.9±0.9[C]
QT-interval (ms)	24.9±1.6	25.3±1.8	26.9±2.0	27.6±2.2
QTm-interval ms)	19.3±1.3	19.8±1.5	22.1±1.7	22.8±1.8

[A]p< 0.05 compared to age- and sex-matched genotype. N=3-5 in each group.
[B]p=0.09. [C]p=0.12, QTm= Murine heart-rate corrected QT-interval.

Supplementary Table S5. Surface ECG parameters
Controls were either Myh6Cre-cap2 $^{+}$/ cap2 $^{+}$, cap2 ^{loxp}/cap2 ^{loxp}, Myh6Cre-cap2 $^{+}$/ cap2 ^{loxp}, or cap2 $^{+}$/cap2 ^{loxp}

	Males: 15 week-old		Females: 15 week-old	
Cap2 genotype	Control	*Myh6Cre-cap2^{loxp}/cap2^{loxp}*	Control	*Myh6Cre-cap2^{loxp}/cap2^{loxp}*
IVS (µm)	680 ± 80	690 ± 90	690 ± 90	700 ± 130
LVPW (µm)	690 ± 70	710 ± 100	700 ± 80	710 ± 110
LVIDd/BW (µm/g)	190 ± 20	210 ± 20	170 ± 20	180 ± 20
LVIDs/BW (µm/g)	130 ± 10	150 ± 20	140 ± 10	150 ± 10
LVEF (%)	54 ± 3.7	51 ± 5.6	61 ± 5.5	60 ± 4.8

[A]p< 0.05 compared to age- and sex-matched genotype. N=3-5 in each group.
IVS = intraventricular septum; LVPW = left ventricular posterior wall; LVIDd = left ventricular internal dimension at diastole; left ventricular internal dimension in systole; left ventricular ejection fraction. BW = body weight.

Supplementary Table S6. Echocardiographic parameters
Controls were either Myh6Cre-cap2 $^{+}$/ cap2 $^{+}$, cap2 ^{loxp}/cap2 ^{loxp}, Myh6Cre-cap2 $^{+}$/ cap2 ^{loxp}, or cap2 $^{+}$/cap2 ^{loxp}
